# Supplementary material for: multiSero: open multiplex-ELISA platform for analyzing antibody responses to SARS-CoV-2 infection
Source: medRxiv. 2021 May 11:2021.05.07.21249238. Preprint. [Version 1] doi: 10.1101/2021.05.07.21249238 (PMC8132273; doi:10.1101/2021.05.07.21249238)
Supplement: 1 [file NIHPP2021.05.07.21249238V1-supplement-1.pdf]

medRxiv preprint doi: https://doi.org/10.1101/2021.05.07.21249238; this version posted May 11, 2021. The copyright holder for this preprint **(which was not certified by peer review)** is the author/funder, who has granted medRxiv a license to display the preprint in perpetuity.
It is made available under a CC-BY 4.0 International license .

Supplementary Figures

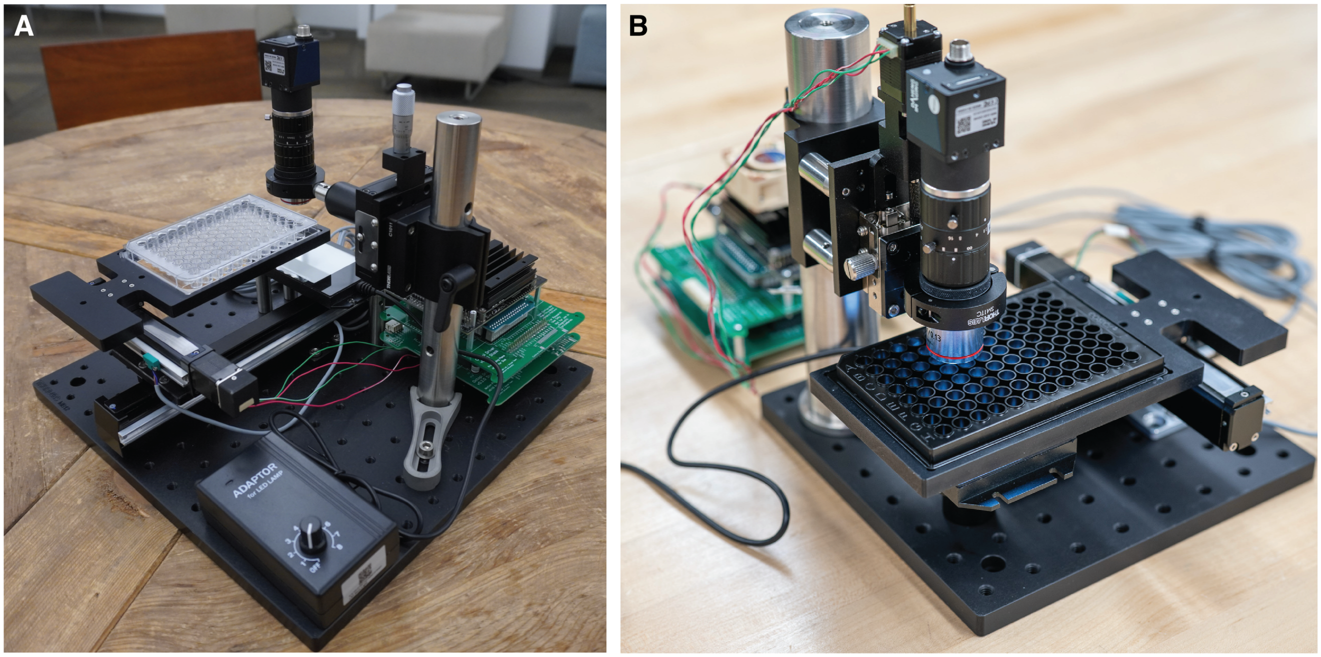

Fig. S1. Photos of Squid plate reader (Nautilus). (A) Photo of the original unit used for data collection. **(B)** Photo of the current version that has motorized focus adjustment.

medRxiv preprint doi: https://doi.org/10.1101/2021.05.07.21249238; this version posted May 11, 2021. The copyright holder for this preprint (which was not certified by peer review) is the author/funder, who has granted medRxiv a license to display the preprint in perpetuity.
It is made available under a CC-BY 4.0 International license .

Tables

#	Description	Vendor	Part Number / File for CNC machining	Qty	Unit Price	Image Reference
	**Motorized Focus Assembly**					
1	ball bearing linear stage, stainless steel, ±6.5mm	dg-sl	LBX40-C (product page)	1	$61.14	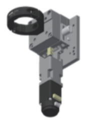
2	adapter for mounting SM1TC to LBX40-C	XTJ-tech	adapter_LBX40_SM1TC(thick)_v2.zip	1	$25.00	
3	adapter for mounting LBX40-C to C1511	XTJ-tech	adapter_LBX40_C1511_v3.zip	1	$25.00	
4	adapter for mounting 21H4U to LBX40-C	XTJ-tech	adapter_LBX40_21H4U_v3.stp	1	$55.00	
5	size 8 captive linear actuator, 6e-5 in/step	Haydon kerk	21H4U-2.5-A98 v1 (based on 21H4U-2.5-907)	1	$130.45	
7	Clamp for SM1 Lens Tubes	Thorlabs	SM1TC	1	$45.72	
8	SM1 Lens Tube, 0.3" threaded depth	Thorlabs	SM1L03	1	$12.52	
9	SM1 adapter for Objectives with RMS thread (Olympus,	Thorlabs	SM1A3	1	$18.50	
11	M3 x 15 mm (or 14 mm) socket head screws for mounti	McMaster-Carr	91292A346 or 91292A027	4		
12	M3 x 5 mm socket head screws for mounting the LBX40	McMaster-Carr	91292A110	4		
13	8-32 x 1/2" socket head screws for mounting the assem	McMaster-Carr	92196A194	4		
14	8-32 x 1/4" socket head screws for mounting SM1TC	McMaster-Carr	92196A190	1		
15	M2.5 x 8 mm socket head screws for adapter mounting	McMaster-Carr	91292A012	2		
16	M2 x 6 mm socket head screws for mounting the linear actuator			4		
			Total: $312.19			
	**"Microscope Body"** (may be replaced by custom machined blocks)					
1	Ø1.5" Post Mounting Clamp, 2.50" x 2.50"	Thorlabs	C1511	1	$71.57	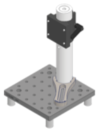
2	Ø1.5" Mounting Post, 1/4"-20 Taps, L = 8" (other length	Thorlabs	P8	1	$59.79	
3	Studded Pedestal Base Adapter, 1/4"-20 Thread	Thorlabs	PB4	1	$13.74	
4	Clamping Fork for Ø1.5" Pedestal Post or Post Pedesta	Thorlabs	PF175B	1	$17.00	
5	Aluminum Breadboard 6" x 6" x 1/2", 1/4"-20 Taps (othe	Thorlabs	MB6	1	$51.48	
6	Ø18.0 mm Sorbothane Feet, Adhesive Mounting Surfac	Thorlabs	AV3	1	$19.91	
7	1/4-20 socket head screw and washer			1		
			Total: $233.49			
	**Imaging Lens and Camera Assembly**					
1	USB3 camera, Sony IMX 226, 1.85 um, 12 MP, 32 fps	Daheng	MER-1220-32U3M	1	$260.00	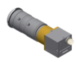
3	f = 50 mm machine vision lens (1/1.8", f2.4, 10 MP ratec	HIKROBOT	MVL-HF5024M-10MP (distributor link, MTF)	1	$136.00	
5	M27 ext - SM1 int adapter for MVL-HF5024M-10MP	Thorlabs	SM1A35	1	$21.86	
			Total: $417.86			
	**Alternative 140 mm x (up to) 140 mm stage**					
1	motorized translation stage, 80 mm travel	Kgg-robot	~~SSMD20-R02-080L~~ (being replaced by alternative from Kgg-robot o	1	$150-800, c	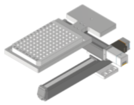
2	motorized translation stage, 140 mm travel	Kgg-robot	~~MVD30-RE18-200L~~ (being replaced by alternative from Kgg-robot o	1	$150-800, c	
6	well plate holder (MIC-6 Al)	XTJ-tech	(being revised)	1	$80.00	
7	well plate format glass slide holder (4 slots)	XTJ-tech	(being revised)	0	$18.00	
			(Total: $400-$1000, depending on options)			
	**LED matrix illuminator**					
1	APA102-2020 8x8 RGB LED Grid	Adafruit	3444 *(to be replaced with custom made board)*	1	$24.95	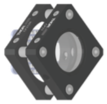
2	Cage Plate, for mounting the LED matrix	Thorlabs	CP37	1	$19.91	
3	Cage Plate, for condenser	Thorlabs	CP33	1	$16.89	
4	Aspheric Condenser Lens w/ Diffuser, Ø25 mm, f=20.1	Thorlabs	ACL2520U-DG6-A	1	$30.84	
5	Cage Rods 1" (pack of 4), for connecting the assembly	Thorlabs	ER1-P4	1	$19.77	
			Total: $112.36			
	**Objective**					
1	4x/0.13 (0.17 coverslip correction) Plan Fluor WD 16.3n	Boli Optics	FM13013231	1	$88.98	
	**Filter**					
1	655 nm/40 nm band pass filter	Semrock	FF02-655/40-25	0	$345.00	
	655 nm/30 nm band pass filter	Chroma	AT655/30m	1	$200.00	
			Total (without motorized XY stage): $1426.02			

Table S1. Nautilus Bill of Materials

medRxiv preprint doi: https://doi.org/10.1101/2021.05.07.21249238; this version posted May 11, 2021. The copyright holder for this preprint
(which was not certified by peer review) is the author/funder, who has granted medRxiv a license to display the preprint in perpetuity.
It is made available under a CC-BY 4.0 International license .

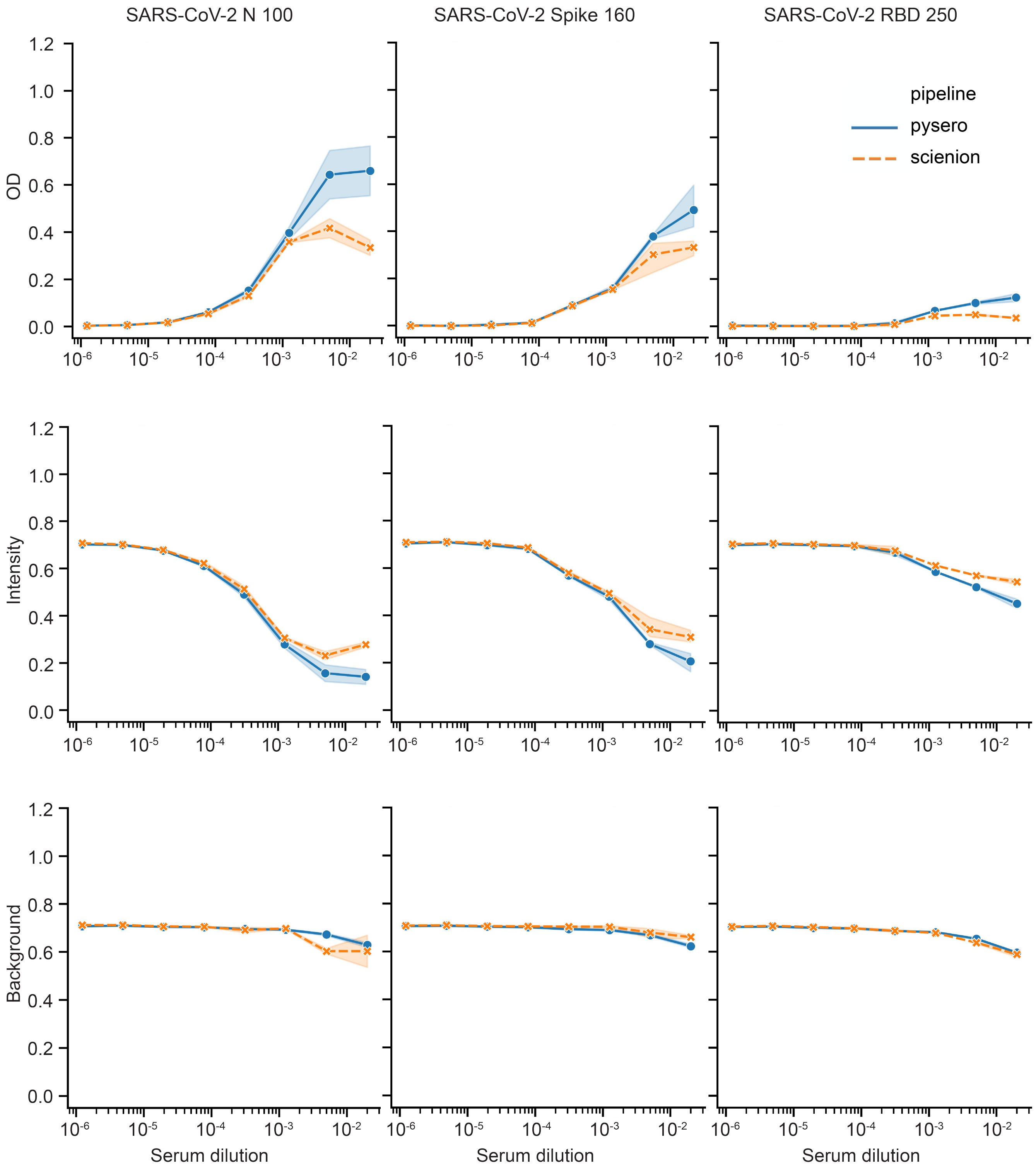

Fig. S2. Comparison of Scienion analysis platform and pysero. Images from a SciREADER CL2 were analyzed using either pysero (blue line) or Scienion analysis platform (orange dashed line). Analyzed OD (top), intensity (middle), and background intensity (bottom) for antibody responses of a single SARS-CoV-2 positive serum to three antigens (left to right: SARS-CoV-2 N, Spike, RBD) are shown as a function of serum dilution. Shades around lines represent 95% confidence intervals around the mean of triplicate spots.

medRxiv preprint doi: https://doi.org/10.1101/2021.05.07.21249238; this version posted May 11, 2021. The copyright holder for this preprint (which was not certified by peer review) is the author/funder, who has granted medRxiv a license to display the preprint in perpetuity.
It is made available under a CC-BY 4.0 International license .

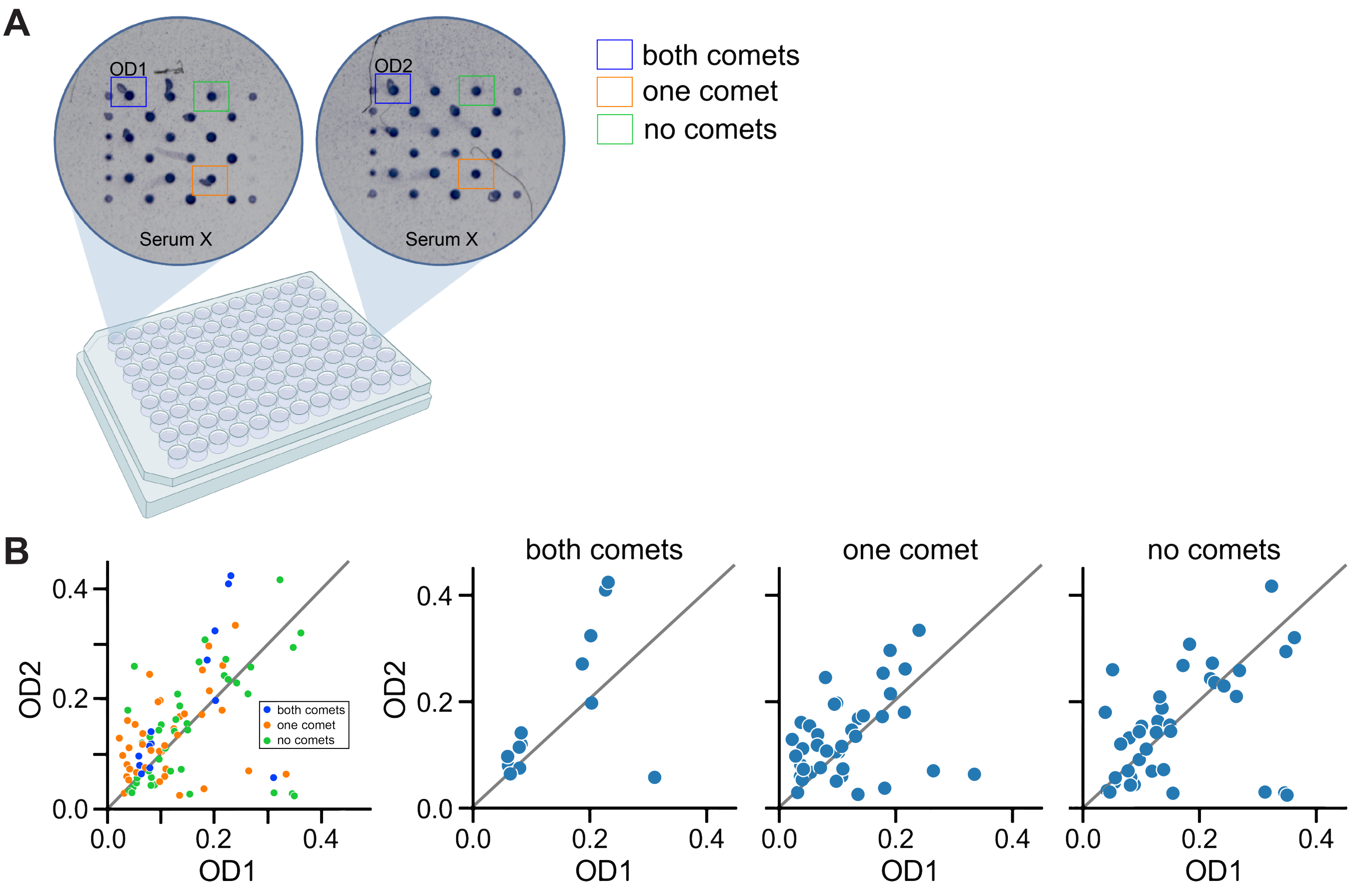

Fig. S3. Evaluation of how comets affect measured ODs using duplicate ELISA-array wells. ODs from spots at the same locations in the array grid were compared across duplicate wells. **(A)** Schematic of example spot-spot comparison. Top-left non-fiducial spot in well A1 containing serum X was compared to the top-left non-fiducial spot in well F12 also containing serum X. **(B)**The data for one plate of duplicate sera are split according to the number of comets in the spot pairs: spot pairs in which one spot had a comet (orange); spot pairs in which both spots had comets (blue); and spot pairs in which neither spot had a comet (green). A y = x line is denoted on the plot (grey line). We find that the presence of comets does not add observable bias or variance to OD measurements.

medRxiv preprint doi: https://doi.org/10.1101/2021.05.07.21249238; this version posted May 11, 2021. The copyright holder for this preprint (which was not certified by peer review) is the author/funder, who has granted medRxiv a license to display the preprint in perpetuity.
It is made available under a CC-BY 4.0 International license .

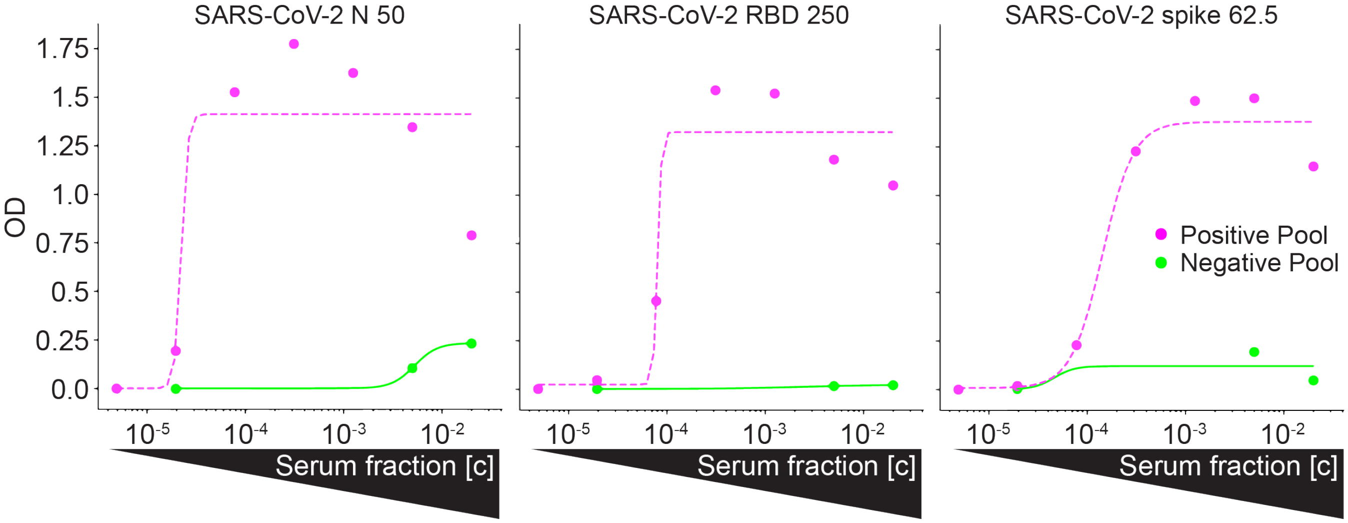

Fig. S4. Reactivity of Positive Pool and Negative Pool. After OD extraction from the registered grid coordinates, pysero generates plots according to user-specified parameters. Representative plots of antibody responses against the 3 SARS-CoV-2 antigens in the array: N (left), RBD (center), and spike (right). Positive Pool indicates pooled sera from SARS-CoV-2 RT-PCR-positive individuals (magenta points) and Negative Pool indicates pooled sera from individuals prior to the pandemic (green points). The respective curves are 4 parameter logistic regressions fit to OD values of a serial dilutions of the sera, with diminished OD values at highest concentrations likely due to the 'hook effect' (8). Pysero can output curve fits (shown above), categorical plots, and receiver operating characteristic analysis.

Antigen	AUC	TPR 95% Specificity	TPR 99% Specificity	Mean +- sd OD of Neg Pool	Mean +- sd [Ab] of Neg Pool
N 50	0.969	0.907 (0.816-0.939)	0.638 (0.398-0.826)	0.1047 ± 0.02238	0.0011 ± 0.00055
N 100	0.968	0.907 (0.822-0.943)	0.541 (0.238-0.834)	0.1087 ± 0.02432	0.0079 ± 0.00036
RBD 250	0.983	0.977 (0.957-0.992)	0.977 (0.936-0.992)	0.0160 ± 0.01834	0.0004 ± 0.00025
RBD 500	0.982	0.977 (0.957-0.992)	0.977 (0.957-0.992)	0.0230 ± 0.00735	*
Spike 62.5	0.994	0.988 (0.973-1)	0.984 (0.905-0.994)	0.1933 ± 0.04399	0.0134 ± 0.00266
Spike 125	0.989	0.984 (0.969-0.996)	0.981 (0.897-0.992)	0.0927 ± 0.01977	*

Table S2. Summary statistics for antigens in ELISA-array. Area under the curve (AUC), sensitivity (true positive rate; TPR) at 95% and 99% specificity (N=507), mean OD and mean binding of Negative Pool expressed as relative concentration of mAb CR3022 standard curve in Negative Pool ([Ab] of Neg Pool; units are $\mu g\ mL^{-1}$) for each antigen. Asterisks in the relative antibody concentration of Negative Pool column indicate that the mean OD of the Negative Pool for these antigens was lower than the lowest mAb CR3022 concentration in the standard curve.

medRxiv preprint doi: https://doi.org/10.1101/2021.05.07.21249238; this version posted May 11, 2021. The copyright holder for this preprint
(which was not certified by peer review) is the author/funder, who has granted medRxiv a license to display the preprint in perpetuity.
It is made available under a CC-BY 4.0 International license .

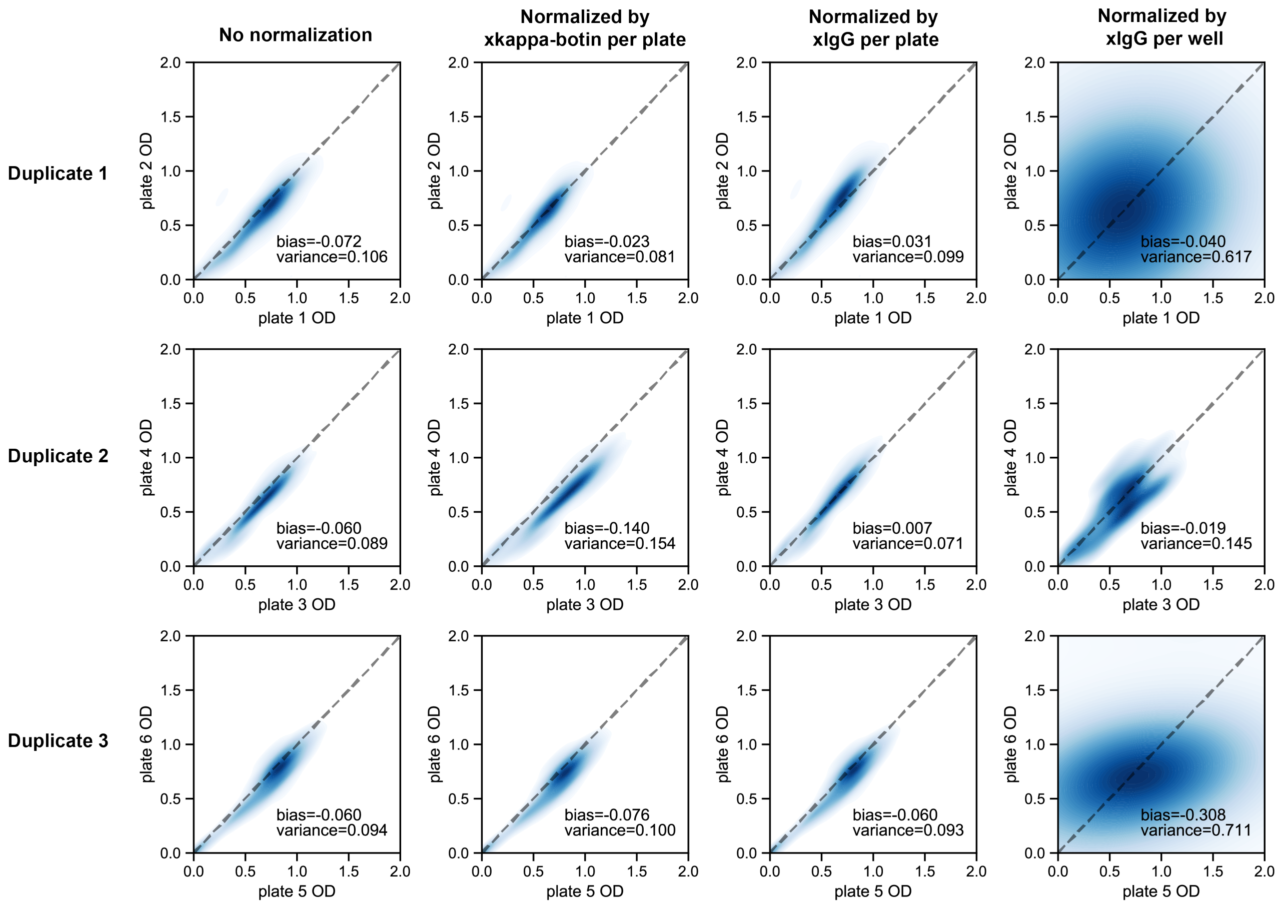

Fig. S5. Comparison of normalization methods for correcting biases and variances in ODs across plates. 2D distribution of duplicate OD values of antibody responses of RT-PCR positive sera to SARS-CoV-2 antigens. Spots with the same array location on duplicate plates are plotted against each other and then smoothed by the kernel-density function to show the density of the data points (indicated by the brightness of the blue color, high brightness indicates low density). The spot OD values were normalized by dividing the mean of the reference spot ODs (anti-IgG Fc or fiducial spot) over each plate or well as indicated at the top. Duplicates with identical spot OD values will follow the function y = x (dashed line). Performance of different normalization schemes are quantified by bias and variance of the normalized OD values across plates, which are defined by mean of y – x and |y – x|. 3 duplicates (6 plates) are shown. Normalizing ODs by the mean of anti-IgG Fc ODs over each plate consistently reduces bias and variance in all 3 duplicates.

medRxiv preprint doi: https://doi.org/10.1101/2021.05.07.21249238; this version posted May 11, 2021. The copyright holder for this preprint (which was not certified by peer review) is the author/funder, who has granted medRxiv a license to display the preprint in perpetuity.
It is made available under a CC-BY 4.0 International license .

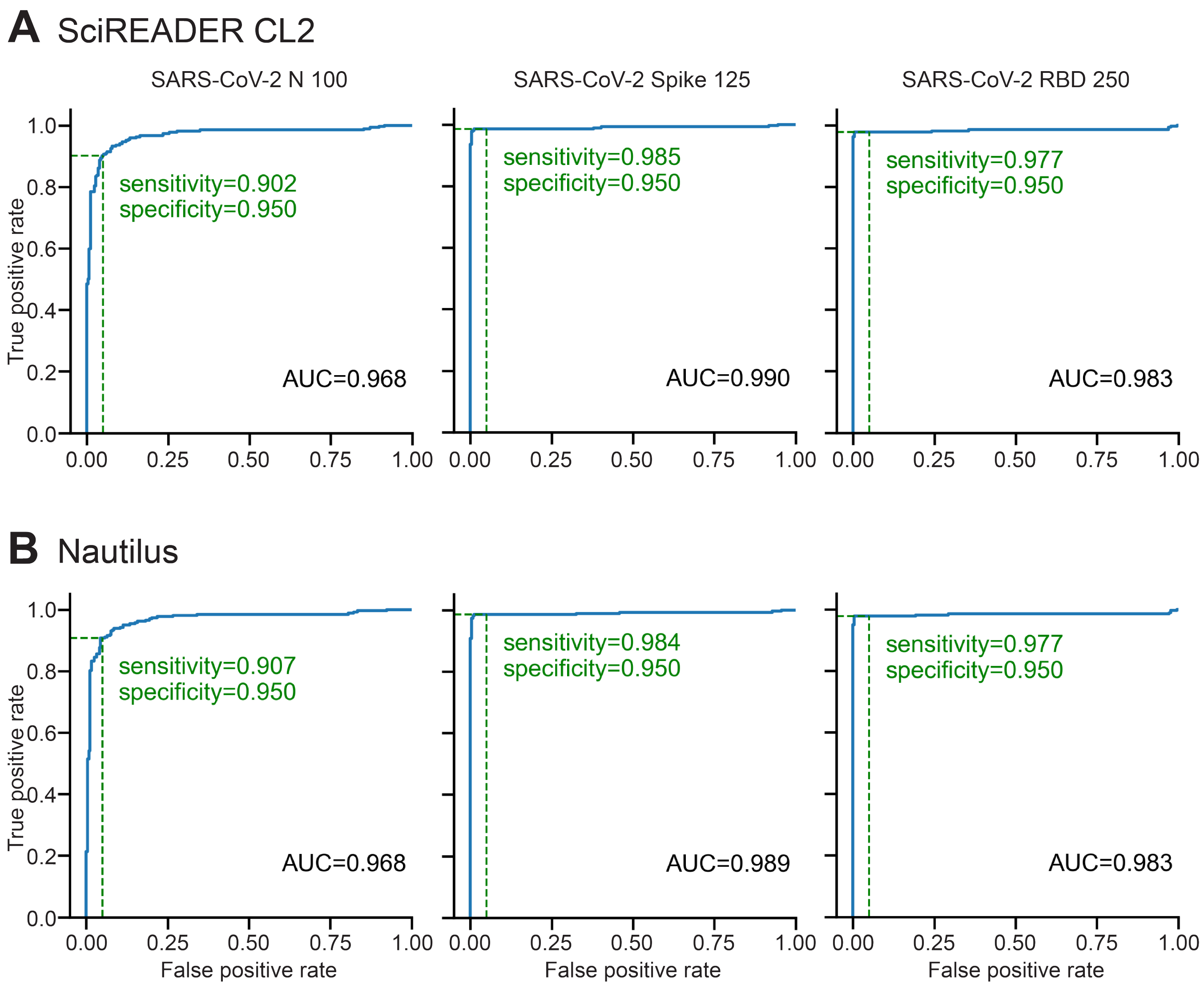

Fig. S6. Classification accuracy for images acquired with SciREADER CL2 and Nautilus, and analyzed with pysero. (A-B) ROC curves for single antigens (left to right: SARS-CoV-2 N, Spike, RBD) for array images acquired using SciREADER CL2 (top) or Nautilus (bottom). Images from each platform were analyzed using pysero. Blue shades represent 95% confidence intervals of ROC curves (blue line). For each antigen, the sensitivity (True positive rate) at 95% specificity (1 - false positive rate) is denoted (green dashed line) on the curve. 95% confidence interval of area under the curve (AUC) is reported below each curve (N=507).
